# Operational and Performance Experience with uMI550 Digital PET-CT during Routine Quality Control Procedures

**DOI:** 10.1055/s-0043-1777696

**Published:** 2023-12-26

**Authors:** Manoj Kumar Singh, V. Sai Krishna Mohan, Chanchal Kaushik

**Affiliations:** 1 Medikabazaar, Technopolis Knowledge Park, Mumbai, Maharashtra, India; 2 Medicover Cancer Institute, Nellore, Andhra Pradesh, India; 3 School of Health and Society, University of Salford, Manchester, United Kingdom

**Keywords:** PET, PET-CT, daily and weekly QC, Ge-68, ^18^ F-FDG

## Abstract

**Introduction** The quality control (QC) procedures for positron emission tomography (PET) scanners are covered by National Electrical Manufacturers Association and International Electrotechnical Commission. QC must be carried out at regular intervals according to the specifications of the scanner manufacturer. Daily and weekly QC plays a valuable role in monitoring positron emission tomography (PET) scanner performance changes. This study shares operational and performance experience of QC procedures that do not require a radioactive Ge-68 source to perform daily QC and experience with fluorodeoxyglucose F18 ( ^18^ F-FDG) as a substitute for germanium-68/sodium-22 (Ge-68/Na-22) source for weekly QC.

**Method** This study was performed on an uMI550 digital positron emission tomography-computed tomography (PET-CT) scanner. In this scanner daily QC checks system temperature and humidity, system count rate, data link status, and voltage. QC was performed at the console control, the position of the scanner table was in the home position pulled out from the gantry, and the room was closed during the quick QC. Weekly full QC check items include look-up table drift, energy drift, time-of-flight status, C-map status, temperature and humidity, and voltage. Weekly full QC was performed with a ^18^ F-FDG source in a rod phantom source.

**Results** Over 200 daily QC tests without a radioactive source Ge-68 phantom and 50 full weekly QC tests using a ^18^ F-FDG rod phantom were performed with this scanner according to the manufacturer's instructions and a test report was generated. No daily QC errors or warnings were observed during this period.

**Conclusion** The new approach for the daily PET QC does not expose operators to radiation. This translates into commercial and operational merits with consistent performance and results.

**Implications for Practice** Reduction in radiation exposure to operating staff during QC procedure in PET-CT scanner.

## Introduction

### Digital PET-CT

The performance of positron emission tomography-computed tomography (PET-CT) scanners has improved significantly over time, one of the reasons being the integration of digital silicon photomultiplier (SiPM) detector technology. 1 2 Other advances include improving of the axial field of view coverage, advances in time-of-flight (ToF) technology, spatial information through optimization of crystal size, and other performance parameters. This hardware upgrade results in an improvement in image quality, timing resolution, sensitivity, effective sensitivity, and reduction in scan acquisition time as well as optimization of patient radiation dose. 1 2 3 4 5 Fluorodeoxyglucose F-18 ( ^18^ F-FDG) has been the radiopharmaceutical used in most PET scans. But now along with the advances in PET scanner technology, the variety of radiopharmaceuticals used for diagnosis has also increased, leading to different types of imaging procedures by PET-CT. These include ^68^ Ga-PSMA, ^68^ Ga-DOTATATE, ^18^ F-DOPA, ^18^ F-PSMA, ^18^ F-Choline, ^18^ F-NaF, and many more. 6 7 8 9 10

### Quality Control Procedures in Digital PET-CT

The quality control (QC) procedures for PET scanners are covered by the NEMA (National Electrical Manufacturers Association), the International Electrotechnical Commission (IEC), 11 12 and manufacturer standards as minimum requirements. The global agencies and NEMA establish standards for imaging testing and procedural guidelines recommendations for all manufacturers for the evaluation of PET scanner performance parameters and the outcomes. These guidelines have also evolved over time with PET technology and performance evolution. 13 This initial PET scanner performance evaluation is used to establish a baseline of measurements, and then the periodic assessment of the scanners' system performance is achieved on an annual, semi-annual, quarterly, weekly, and daily basis. 14 These QC must be carried out at regular intervals according to the specifications of the scanner manufacturer. 15 16 This QC plays a critical role in reproducing accurate diagnostic results. Daily, weekly, quarterly, half-yearly, and later periodic Q play a valuable role in monitoring PET system performance stability and changes. Any issues and malfunctions encountered during QC procedures must also be recorded, reported, and resolved in order to perform a prior clinical scan. 17 18

### QC Radioactive Sources in PET-CT

The most commonly used radioactive long-life sealed sources are germanium-68 (Ge-68)/sodium-22 (Na-22). It is compatible and used by most PET-CT scanners. The Ge-68 and Na-22 QC sources are available in different types such as point source, annulus phantom, and cylindrical source. In most PET-CT scanners, the quality assurance (QA) process is performed with a sealed cylindrical Ge-68 radioactive source to ensure optimal PET reproducibility. 19 20 21 Ge-68 is used as a long half-life PET source for attenuation correction and calibration of PET scanners. 22 The half-life of Ge-68 is 270.95 days, decay by electron capture gamma and X-rays: 10.3 keV (46%), 9.25 keV (25.6%), 9.22 keV (13.1%), to the daughter radionuclide gallium-68. Ge-68 is commonly used as the cylindrical and annulus phantom of Siemens and GE PET-CT scanners, respectively. Na-22 with a half-life of 2.6 years, decay by positron (90.2%, 1.27MeV), and electron capture (9.7%) are used as a point and disc source by Philips PET-CT scanners. 22 23 24 The annulus phantom developed for GE Healthcare's PET-CT scanner is packed with Ge-68 radionuclide with a radioactivity range of 37-55 MBq (1-1.5 mCi) and the source lifetime is 2 years for replacement and optimal performance to reproduce precise results. Similarly, the Siemens Healthineers cylindrical phantom is also filled with Ge-68 radionuclide with a radioactivity range of 74 to 92.5 MBq (2–2.5 mCi) and a source lifetime of 1 to 2 years. Philips healthcare PET-CT scanners are designed for point sources of Na-22 with an activity of 3.7MBq and one of the lowest radioactivity with a lifetime of 2 years. 24 These radioactive sources used for QC procedures contribute to radiation exposure of personnel. 25

### uMI550 Digital PET-CT

The uMI550 digital PET-CT system (Shanghai United Imaging Healthcare, China) is one of the first digital PET-CT scanners designed considering the current challenges of radioactive Ge-68 phantoms on a global scale with the latest technology integrated into SiPM and hardware to meet the minimum QC requirements. 26 The design and technology of this uMI550 PET-CT system have transformed the daily QC methodology, where the daily QC can be performed without using the Ge-68 radioactive source phantom, and in the weekly QC, this system has been further developed to use the radioactivity-based QC using ^18^ F-FDG or ^18^ F-sodium fluoride ( ^18^ F-NaF) uses as an alternative option. In the standard and traditional practice in PET-CT imaging, be it analog or digital PET-CT systems, QC of the PET system must be ensured prior to the clinical scan to ensure the accuracy of the quantitative and qualitative analysis of standard uptake values (SUV) measurements and to ensure reproducibility in oncological and nononcological treatments. Most PET-CT models are manufactured by Siemens Healthineers, GE Healthcare, Philips Healthcare, Neusoft, and Canon Medical. 24 25 These systems rely on daily QC using long-lived Ge-68 or Na-22 radionuclides. These systems are limited by the daily and periodic QC reliability of the Ge-68 cylindrical phantom source. The main challenges related to a radioactive source are (a) the cost of procuring the shielded radioactive source, (b) the lifetime of this source is maximum 2 years and needs to be replaced every 2 years, (c) safety and shielding requirements due their radioactive nature in accordance with local and national regulations, (d) exposure to radiation from daily bare-handed handling, (e) the cost of returning the expired source to the United States of America/Europe, and the country's state and (f) regulatory permitting documentation with each procurement or return and maintenance of these records. Due to unavoidable circumstances at the international or national level, there will be delays in providing sources in a timely manner, and without this PET-CT system, it cannot be used for clinical scans and will affect the patient's diagnosis and treatment overall. With continued advances in technology and attempts to reduce radiation dose to personnel, it is time to examine the efficacy of employing a nonradioactive source or alternate technical method to perform daily QC procedures. Using an alternate technical method that does not require any radioactive source for QC procedures is essential not only in terms of completely eliminating radiation exposure to personnel during daily QC but also in reducing departmental and logistics costs. This study is nonetheless a valuable contribution to the field as it shares operational and performance experience with uMI550 digital PET-CT during routine QC procedures that do not require a radioactive Ge-68 source to perform daily QC and experience with ^18^ F-FDG as a substitute for Ge-68/ Na-22 source for weekly QC.

## Materials and Methods

This study was conducted using the uMI550 digital PET-CT scanner installed at Medicover Cancer Institute Nellore Andra Pradesh, India in 2020. The QC data were recorded and analyzed from December 2020 to March 2022. This is one of the initial installations of United Imaging Healthcare's uMI550 digital PET-CT system in the country.

### uMI550 Digital PET-CT Scanner

This system is equipped with ToF and SiPM detector technology. The scanner features a 24 cm wide axial field of view embedded in a 2.76 × 2.76 mm lutetium yttrium oxyorthosilicate (LYSO) crystal, paired with a SiPM detector and integrated into a 40 physical rows CT scanner and a 2.2 cm wide CT detector cover. 26 It offers some unique operational advantages such as not requiring a radioactive source to perform the daily QC test and therefore reducing the total cost of operation and downtime as it does not reply on long life Ge-68 source phantoms. This reduces daily handling of Ge-68 radioactive sources compared to other digital and traditional analog PET-CT scanners. For weekly QC testing, this system also requires a radioactive source for QC testing, as many QC performance parameters always require the radioactive source to verify performance. The uMI550 scanner is designed and integrated with ^18^ F-based radionuclide data in QC operation instead of Ge-68/Na-22 to support and be compatible with ^18^ F-based radionuclides. These unique features result in similar operational stability benefits without additional operational costs, and reduce radiation exposure from 7 days to once a week. This is a new concept and method of QC in PET-CT imaging. The durability and stability of this system are observed prospectively and this method is also compared with other PET-CT scanners. The system tolerance limit and QC test parameters are listed in Table 1 .

**Table 1:** Daily and weekly QC tests parameters and tolerance limits

Imaging system	Frequency	Radioactive source	Test	Procedures	Tolerance criteria
PET	Daily	No	Count rate (kcps)	Detect and check the count of random and scatters without radioactive source	25.0–160.0
	Daily	No	Temperature	System initialization and temperature measurement at hardware of detector and SiPM for operational condition	14.0–37.0
	Daily	No	System humidity	Checking humidity level within room and at hardware assembly for safe operation of detector	30.0–70.0
	Daily	No	Voltage	Checking for stable and constant voltage for SiPM safe operation within limit	33.00–37.00
	Weekly	Yes	LUT drift	PET rod phantom filled with ^18^ F-FDG, positioned to perform QC for LUT drift	0.00–0.46
	Weekly	Yes	Energy drift	PET rod phantom filled with ^18^ F-FDG, positioned to perform QC for energy drift	0–8
	Weekly	Yes	TOF status	PET rod phantom filled with ^18^ F-FDG, positioned to perform QC for TOF status	0.00–30.00
	Weekly	Yes	CMap status	PET rod phantom filled with ^18^ F-FDG, positioned to perform QC for CMap status	0–5
	Weekly	Yes	Temperature	System initialization and temperature measurement at hardware of detector and SiPM for operational condition	14.0–37.0
	Weekly	Yes	System humidity	Checking humidity level within room and at hardware assembly for safe operation of detector	30.0–70.0
	Weekly	Yes	Voltage	checking for stable and constant voltage for SiPM safe operation within limit	33.00–37.00

Abbreviations: ^18^ F-FDG, fluorodeoxyglucose F18; LUT, look-up table; PET, positron emission tomography; QC, quality control; SiPM, silicon photomultiplier; TOF, time of flight.

### Comparison with Other PET-CT Systems

The daily and weekly QC of the uMI550 system was also compared with another manufacturer of PET-CT systems. The comparison includes the radioactive source requirement for QC and, frequency of QC with radioactive and without radioactive sources (QC parameters checks and workflow as shown in Table 2 ).

**Table 2:** Comparison with other PET-CT scanners

Test	Frequency	QC requirements	Siemens PET scanners	GE PET scanners	Philips PET scanners	GE PET scanners	United Imaging PET scanners
			Valladares et al 2019 24	Valladares et al 2019 24	Valladares et al 2019 24	Hallab 2022 25	Present study
Sealed QC Source	Daily/Weekly	PET image quality and quantitative analysis reproducibility	^68^ Ge cylindrical phantom sealed source	^68^ Ge PET annulus phantom sealed source	^22^ Na-point and disc sealed source	^68^ Ge PET annulus phantom sealed source	No Radioactive source for daily QC/weekly QC with ^18^ F-FDG
Daily QC	Daily	To initialize the hardware test and confirm the operational status of the PET detectors and connected electronics, temperature, humidity, voltage, count rate, and, and system sensors for optimal performance within the limits specified by the manufacturer	^68^ Ge/ ^68^ Ga cylindrical phantom: Partial PET detector setup Normalization Scatter ratio Block noise Block efficiency E correction factor Sinograms evaluation Measured randoms Image plane efficiency Scanner efficiency (E)	^68^ Ge/ ^68^ Ga PET annulus phantom: Coincidence (sensitivity) Energy peak Singles Deadtime Timing Gain changes	^22^ Na-point and disc source: System initialization Hardware sensor test PMT gain calibration Energy Timing Emission sinogram Baseline collection	^68^ Ge/ ^68^ Ga PET point source: Coincidence (sensitivity) Singles Deadtime Timing Energy peak Gain changes	No radioactive source: System initialization Hardware test Count rate Energy Temperature Humidity Voltage 0 & 1 data path status
Weekly QC	Weekly	Ensuring proper calibration and validation of acquired values by using a radioactive source phantom against a scanner reference value for optimal performance of quantification results	Complete PET detector setup Normalization Sinograms evaluation Block noise Block efficiency Measured randoms Scanner efficiency (E) Scatter ratio E correction factor Image plane efficiency	Coincidence (sensitivity) Singles Deadtime Timing Energy peak Gain changes Sinograms evaluation	System initialization Hardware sensor test PMT gain calibration Energy Timing Emission sinogram Baseline collection	Coincidence (sensitivity) Singles Deadtime Timing Energy peak Gain changes Sinograms evaluation	Inhouse rod Phantom filled with ^18^ F-FDG (1mCi): System initialization Hardware test Count rate Energy drift, LUT drift, sinogram, TOF status and CMap status
Image quality	Annually or major service	Evaluation of the reconstructed PET image for qualitative and quantitative analysis	NEMA IQ phantom: Contrast recovery Background variability	NEMA IQ phantom: Contrast recovery Background variability	NEMA IQ phantom: Contrast recovery Background variability	NEMA IQ phantom: Contrast recovery Background variability	NEMA IQ phantom: Contrast recovery Background variability

Abbreviations: ^18^ F-FDG, fluorodeoxyglucose F18; LUT, look-up table; NEMA IQ, National Electrical Manufacturers Association Image Quality; PET-CT, positron emission tomography-computed tomography; QC, quality control; TOF, time of flight.

### Daily QC Methodology

The daily PET QC test on this uMI550 digital PET-CT system was performed every 24 hours according to the manufacturer's recommendations. This test was performed early each morning before proceeding with patient clinical scans according to department protocols. As mentioned above, according to the manufacturer's recommendation, the daily QC of this scanner does not require a radioactive source. This system is based on a semiconductor-based SiPM detector. The daily QC of the uMI550 system has been designed to focus on important parameters that must be checked daily without using the radioactive source and must be within acceptable limits. Performing the daily QC test was quick and easy and contained only a few steps in protocol. First, the PET-CT table must be in the isocenter with the gantry. There were no requirements for positioning any phantom. The technologist conducts the daily QC procedure from the console room. This daily QC test was quick and took about 10 minutes and generated a result report. For this scanner, the daily QC check parameters are system temperature and humidity as SiPM detectors in the digital PET-CT system take these two into account, the system count rate using the background activity, the data link status, and the voltage stability of SiPM. Since no radioactive source is required, therefore there is no radiation exposure to the operator.

### Weekly Rod Phantom and QC Methodology

The uMI550 digital PET-CT requires the weekly QC with a radioactive source similar to other manufacturers' PET-CT systems as shown in Table 2 . This scanner was QC compatible with ^18^ F-FDG ( ^18^ F-based radionuclide), eliminating operational costs and downtime. During the weekly QC test, the system checks the key parameters that need to be calibrated with a radioactive source, and these parameter values need to undergo QC once a week. This QC test requires (a) the preparation of rod phantom, (b) the positioning of QC phantom on the scanner table, (c) the QC procedure command on the console system, (d) the QC report, (e) and removing the rod phantom source.

### Rod Phantom Preparation

**Fig. 1:**
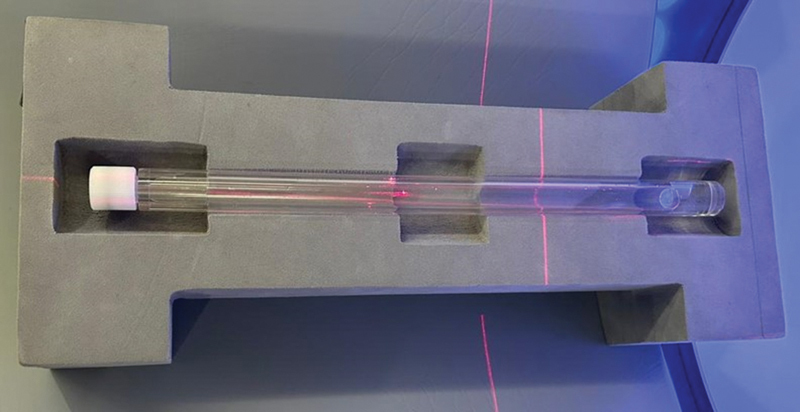
Rod Phantom for weekly quality control.

This system was equipped with a compact rod phantom to perform weekly QC with ^18^ F-FDG as an alternative to the standard sealed Ge-68 source phantom. The volume capacity of this rod phantom is 80 to 85 mL and this phantom can be manufactured in a PET radiopharmacy laboratory using ^18^ F-FDG/NaF radioactivity. The amount of radioactivity was 37 MBq ± 20% (1 mCi). The rod phantom was prepared and first filled with normal water to about 95% of the volume capacity and about 37 MBq was taken from the ^18^ F-FDG vial behind the lead-shielded L-bench and put into the phantom, later the phantom was closed and the proportions shuffled four to five times, then the remaining space was filled. The preparation time of this phantom was less than 3 minutes and this phantom resulted in approximately nine times less radiation exposure to the technologist when positioning and performing weekly QC compared to a standard whole-body PET-CT scan in which the patient was injected with 370 MBq (10 mCi). Rod phantom is shown in Fig. 1 .

### Weekly QC Procedure

The rod phantom was prepared in radiopharmacy laboratory. After preparation, the technologist brought the phantom into the scanning room and positioned it on a dedicated bracket on the table according to the instructions in the manual provided by the manufacturer. Positioning of the phantom and scanning of the isocenter with the laser is shown in Fig. 1 . After positioning the phantom in the PET isocenter on the couch, the technologist performed the weekly QC test according to the protocol in the control panel system. The procedures for conducting QC were similar to other manufacturer's systems. In the weekly full QC test, the checking parameters are the look-up table (LUT) drift of the LYSO crystal, detector performance, the energy drift, the ToF status of the time resolution performance, the C-map status, humidity, temperature, voltage, and sinogram map as shown in Table 3 . This weekly QC test took about 20 minutes and a QC report was generated. It checks the acquired values with reference values of the system.

**Table 3:** Weekly quality control results

	LUT drift	Energy drift	TOF status	CMap status	Temperature	System humidity	Voltage 0	Voltage 1	Result
Sl no./Normal range	(0.00, 0.46)	(0,8)	(0.00, 30.00)	(0, 5)	(14.0, 37.0)	(30.0, 70.0)	(33.00, 37.00)	(33.00, 37.00)	P: Pass F: Fail
1	0.1	0	15.8	0	High: 31.3 Low: 27.9	44	34.98	34.98	P
2	0.12	1	15.4	1	High: 31.8 Low: 26.7	38	34.44	34.44	P
3	0.15	0.5	13.8	0	High: 33.3 Low: 21.5	39	34.58	34.58	P
4	0	0.2	14.4	2	High: 30.2 Low: 22.4	42	36.22	36.22	P
5	0.11	0.3	15.4	1	High: 29.7 Low: 26.3	41	38.84	38.84	P
6	0.12	0	15.2	0	High: 33.7 Low: 24.1	47	33.22	33.22	P
7	0.1	0.1	14.8	0	High: 28.5 Low: 20.1	43	34.68	34.68	P
8	0.14	1	14.2	1	High: 34.4 Low: 26.8	44	34.98	34.98	P
9	0.1	0.2	12.8	0	High: 32.2 Low: 20.6	43	34.66	34.66	P
10	0.13	0.2	16.6	1	High: 30.8 Low: 23.5	42	36.42	36.42	P

Abbreviations: LUT, look-up table; TOF, time of flight.

### QC Report

After completing the weekly QC procedures on the console system, the results of the QC report showed pass or fail or warning, and a pdf report was also generated with detailed information on QC parameters. Weekly QC parameter consistency was observed with ^18^ F-FDG over the period. Fluctuations in QC parameters were also noted. A delay in QC operation was also observed due to the radioactivity of the rod phantom. All weekly QC parameters are listed in Table 3 .

After the successful completion of the weekly QC, the radioactive rod phantom was removed from the couch and placed in the lead-protected decay container. The scanner was prepared for clinical use. Since the ^18^ F-FDG radioactivity used for weekly QC was low and had a short half-life, it decayed within a few days and the phantom can be reused after a week to ensure complete decay of ^18^ F-FDG radioactivity.

## Result

**Fig. 2:**
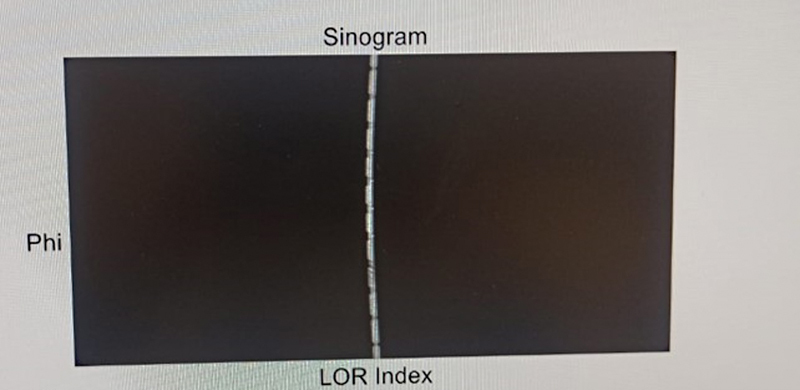
Sinogram.

**Fig. 3:**
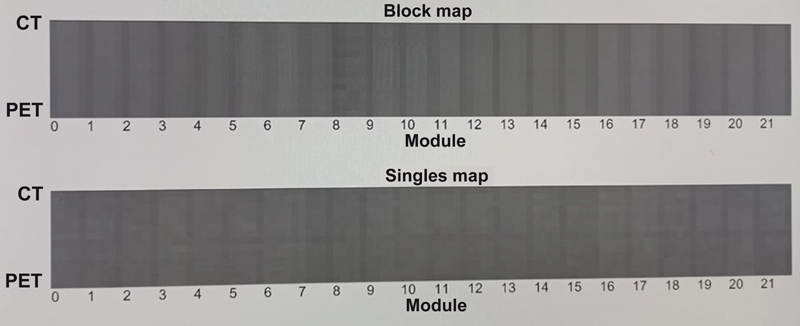
Block image. CT, computed tomography; PET, positron emission tomography.

**Fig. 4:**
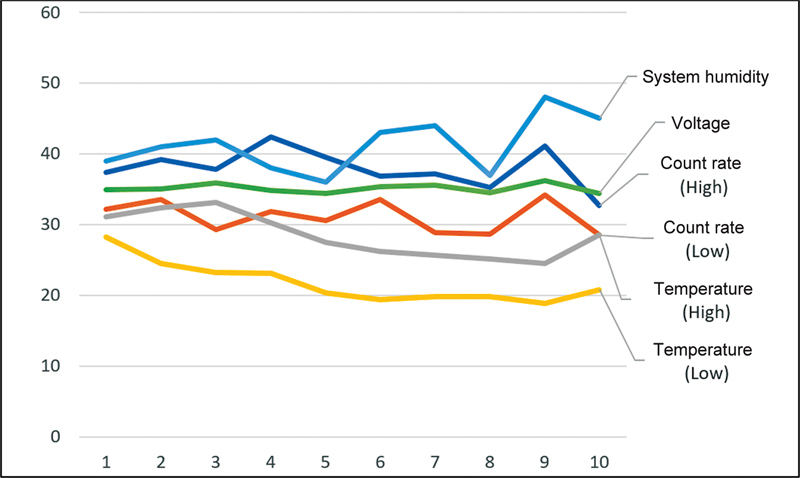
Daily quality control test parameters consistency.

**Fig. 5:**
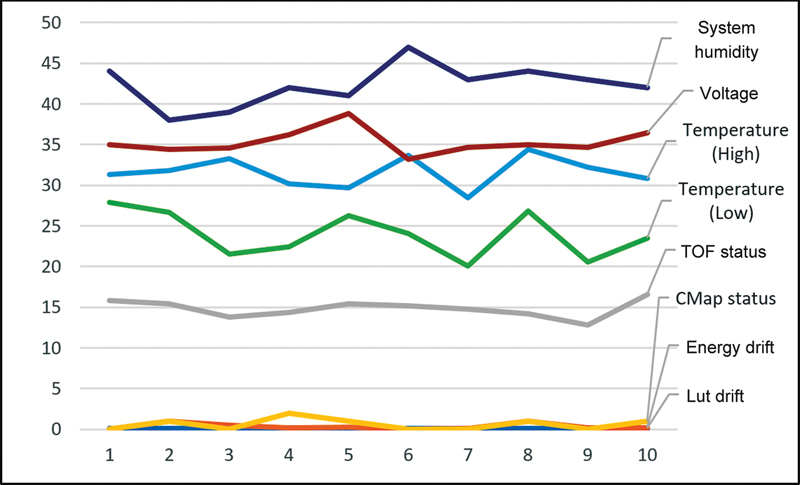
Weekly quality control test parameters consistency. LUT, look-up table; ToF, time-of-flight.

With this scanner, over 200 daily QC tests without a radioactive source Ge-68 phantom and 50 full weekly QC tests using an ^18^ F-FDG rod phantom were performed and a test report was prepared according to the manufacturer's instructions. Table 1 shows the daily and weekly QC test parameters and their tolerance limit according to the manufacturer's system manuals. Table 4 shows the daily QC results and Table 3 shows the weekly QC results. Fig. 1 shows the rod phantom used for weekly QC. Fig. 2 shows a sinogram image and Fig. 3 shows detector block and single map images with a captured rod phantom filled with radioactivity. Figs. 4 and 5 show the consistency of the daily and weekly QC test parameters.

**Table 4:** Daily quality control results

	Count rate (kcps)	Temperature	System humidity	Voltage 0	Voltage 1	Data path	Result
Sl no./normal range	(25.0, 160.0)	(14.0, 37.0)	(30.0, 70.0)	(33.00, 37.00)	(33.00, 37.00)	Status I	P: Pass F: Fail
1	High: 37.4 Low: 32.2	High: 31.1 Low: 28.2	39	34.98	34.98	Status I	P
2	High: 39.2 Low: 33.5	High: 32.4 Low: 24.5	41	35	35	Status I	P
3	High: 37.8 Low: 29.3	High: 33.1 Low: 23.2	42	35.86	35.86	Status I	P
4	High: 42.4 Low: 31.8	High: 30.2 Low: 23.1	38	34.86	34.86	Status I	P
5	High: 39.5 Low: 30.6	High: 27.5 Low: 20.4	36	34.42	34.42	Status I	P
6	High: 36.9 Low: 33.5	High: 26.2 Low: 19.4	43	35.32	35.32	Status I	P
7	High: 37.2 Low: 28.9	High: 25.7 Low: 19.8	44	35.54	35.54	Status I	P
8	High: 35.3 Low: 28.7	High: 25.1 Low: 19.4	37	34.46	34.46	Status I	P
9	High: 41.1 Low: 34.2	High: 24.5 Low: 18.9	48	36.22	36.22	Status I	P
10	High: 32.7 Low: 28.5	High: 28.5 Low: 20.8	45	34.44	34.44	Status I	P

## Discussion

QC of medical imaging equipment is vital to ensure their proper functioning and to attain accurate quantitative results and stability throughout the life of the device. Daily and weekly QC tests play a critical role in monitoring the performance and consistency of the baseline values and their deviation and whether the deviation values are relevant to examine and rectify them at the service level to maintain the baseline values. Routine monitoring of QC results also means the deficiency is identified at an early stage and remediation can be performed without interrupting routine clinical scans.

Over the decades, the daily QC of the PET scanners have been performed utilizing the radioactive source. The radioactive source for daily QC is broadly a sealed source of either Ge-68 or Na-22 and has a half-life of 1 or 5 years. The usage life relies on the minimum radioactivity in the source according to the manufacturer's recommendations for optimal performance and an accurate result. Due to their radioactive nature, these radioactive sources require regulatory and environmental agency approval before procurement and use. There is a substantial amount of documentation, testing, and security to manage these sources during transport or use within the clinical facility. These procedures also place an economic burden on the facility and delays in utilizing the PET-CT clinical scan equipment. 1 16 22 23 24 27 28 Dealing and handling of radioactivity, either for QC or for clinical scans in nuclear medicine, lead to radiation exposure of the operating radiographer or technologist. Performing daily and weekly QC is one of the unavoidable situations where the operator needs to hold the phantom source filled with radioactivity by hand and position it on the PET scanner. This manual handling of radioactivity during the positioning of the phantom in the PET scanner leads to radiation exposure of the operator. In the present study, the manufacturer's established protocols for periodic QC testing were followed and performed. The novel approach of daily QC testing without a radioactive source was performed and the results were recorded for 15 months. Several international bodies and manufacturers have published QC guidelines and recommendations for a PET imaging modality to ensure adequate performance of the systems to achieve accurate qualitative and quantitative outcomes in clinical practice, as well as the frequency of these tests. 1 16 22 23 24 27 28 QC as a segment of a QA program aims to monitor that a system is performing as expected. In order for the PET part to function properly, the detectors and measuring electronics must function properly. By checking detector stability, sudden fluctuations in the detector assembly can be diagnosed early. Review of energy window, LUT, and timing and ToF status results in optimal performance of quantification parameters such as SUV and metabolic tumor volume. Equally important are the other parameters such as temperature, voltage, and humidity work in the optimal range. Optimal SiPM performance depends heavily on temperature, voltage, and humidity. Normalization is one of the most important corrections to overcome the inconsistency between the efficiencies of each detector and maintain the uniformity of the final reconstructed PET images. In the present study, daily QC was carried out using a new technique from the manufacturer. Over 200 daily QC tests were conducted and recorded without using a radioactivity filled Ge-68 phantom or any other radioactive source. No daily QC errors or warnings were observed during this period. All daily QC parameters collected were recorded to be within the range according to the manufacturer's baseline values listed in Table 1 . All recorded values were found to be consistent with baseline. Over 50 weekly QC tests have been performed and recorded using this scanner. The weekly QC of this scanner also requires the radioactivity to perform the QC test. The uMI550 PET-CT scanner is capable of performing weekly QC with ^18^ F-FDG energy, while other manufacturer's scanners are activated using Ge-68 energy. Since this scanner is also compatible with ^18^ F-FDG radionuclides, the manufacturer has provided one dedicated inhouse rod phantom for weekly QC. All weekly QC testing was performed by using this in-house rod phantom. The radioactivity required to perform QC with ^18^ F-FDG is 37 MBq (1 mCi). No weekly QC test failures were observed and reported. All recorded values were consistent and within the range of baseline values according to the manufacturer's equipment manual. When compared with the QC test results of PET-CT scanners from Philips, Siemens, and GE Healthcare, 1 16 24 the QC tests of these scanners were found to be dependent on a Ge-68 and Na-22 source, which must be procured to carry out the QC ( Table 2 ). Any source or phantom use filled with radioactivity must be approved by the national regulatory agency (in India, Atomic Energy Regulatory Board [AERB]) that entails various application procedures and pre-import approvals, as well as financial costs to safely transport the radioactivity. 29 The uMI550 PET-CT scanner comes with an in-house rod phantom and eliminates the need for approval from regulatory and environmental authorities to procure the sealed radioactive source for QC. This results in commercial as well as operational advantages over other PET-CT scanners. In our experience, performance was extremely positive, implementation was easy, and time and money were saved on both daily and weekly QC procedures. These daily QC procedures were compared with Hallab et al 16 they have reported the daily QC procedures of their GE PET-CT scanner using a radioactive Ge-68 point source. This Ge-68 point source was shielded and attached to the back of the gantry with a shield. In QC mode, the point source automatically extends and performs QC. This QC method also does not involve any radiation exposure to operating staff. The limitation was that it used to take up a lot of space in the gantry. This method is no longer available with the latest PET scanners. The latest GE PET scanner's QC was published by Valladares et al. 24 It was reported that PET-QC was performed using a Ge-68 annulus phantom. This QC procedure requires manual handling of the annulus phantom and positioning in the PET scanner to perform daily QC. Similarly, Matheoud et al 1 reported PET-QC in a Siemens PET scanner using a cylindrical Ge-68 phantom. Compared to other studies, the present method was found to be unique in eliminating operator radiation exposure during daily QCs. 1 16 24

### Limitation of Study

This is a single-center study using the uMI550 PET-CT system. Further multicenter studies could be conducted on multiple scanners with comparable performance range.

## Conclusion

This study shares operational and performance experiences with the new approach of the uMI550 digital PET-CT for daily and weekly QC procedures. Daily QC procedures do not require any radioactive source and are simple and quick to perform. Weekly QC with this scanner is performed using an in-house with ^18^ F-FDG source compared to the traditional sealed Ge-68 source. This translates into commercial and operational benefits for the operators and center.

### Implications for Practice

The uMI550 digital PET-CT helps reduce operating cost, reduce downtime as it does not rely on the Ge-68 source; it also reduces radiation exposure to operational personnel during daily QC procedure.
